# Lived experiences of tuberculosis patients accessing treatment during COVID-19 in Zimbabwe

**DOI:** 10.4102/safp.v68i1.6228

**Published:** 2026-01-23

**Authors:** Idah Moyo, Livhuwani Tshivhase, Limkile Mpofu

**Affiliations:** 1 Department of Health Studies, College of Human Sciences, University of South Africa, Pretoria, South Africa; 2 Department of Psychology, College of Human Sciences, University of South Africa, Pretoria, South Africa

**Keywords:** access, COVID-19, experiences, tuberculosis, interpretative phenomenology, tuberculosis care services, Zimbabwe

## Abstract

**Background:**

Despite being a preventable and curable disease, tuberculosis (TB) remains one of the deadliest infectious diseases, responsible for over a million deaths worldwide. Global efforts to eradicate TB were disturbed by the emergence of the coronavirus disease 2019 (COVID-19). Zimbabwe was not exempt from the scourge of this pandemic. Like other healthcare services that were disrupted, TB care services were affected. The objectives of this study were to explore the lived experiences of TB patients as they accessed and continued TB treatment during the COVID-19 period in Zimbabwe.

**Methods:**

An interpretative phenomenological analysis (IPA) was conducted. Fifteen TB patients accessing care at the four most populated primary healthcare facilities in Zimbabwe were purposively sampled and participated in the study. The sample size of 15 participants was determined by data saturation. Interpretative phenomenological analysis steps were followed in the data analysis process.

**Results:**

Three themes emerged: psychological effects (anxiety, fear, unintended disclosure, stigma, discrimination); support systems (healthcare facility, family, community); and TB service delivery gaps (delayed diagnosis, poor follow-up and support, inadequate health education).

**Conclusion:**

The study established that the COVID-19 pandemic affected TB service delivery and support, and follow-up with TB clients was not done. Insights from this study are crucial for strengthening the country’s preparedness and response to future epidemics.

**Contribution:**

To facilitate continuity of TB care services, it is critical for decision-makers to develop context-specific intervention strategies and preparedness plans for use during pandemics and other public health emergencies.

## Introduction

Tuberculosis (TB) is reported to have become the second biggest global cause of annual infectious disease deaths after the coronavirus disease 2019 pandemic (COVID-19).^1^ According to the World Health Organization (WHO), TB remains a significant public-health problem in the WHO African region, accounting for 23% of new cases and 31% of TB-related deaths.^2^ However, the disease can be contained and treated successfully following a treatment regimen. Treatment for clients who are diagnosed and treated is usually initiated and followed up in healthcare facilities until they are discharged following a cure. The WHO^3^ indicates that the COVID-19 pandemic had a severe impact on TB service delivery worldwide. The fact that COVID-19 has similar symptoms to TB complicated the public-health response to both diseases, particularly in healthcare facilities.^4^ In addition, because of the severity of the COVID-19 pandemic, it resulted in the disease being prioritised at the expense of TB service delivery, leading to resources, healthcare workforces, material and finances being redirected to COVID-19 pandemic activities.^5,6^ During the COVID-19 pandemic, pulmonary TB, cardio-respiratory diseases and diabetes were found to be the main comorbidities that worsened the severity of COVID-19.^3^

The COVID-19 pandemic resulted in lockdowns locally, nationally and internationally that made clients face difficulties in accessing healthcare facilities.^7^ Studies conducted in Kenya, Malawi and Zimbabwe reported concerns about the interruption of TB treatment and the lack of quality healthcare services for patients because of the COVID-19 pandemic.^7,8,9^ These studies indicated the negative impact of the COVID-19 pandemic on TB patients. Similarly, in Spain, the pandemic disrupted TB care services, including preventative services, because of the lockdown measures.^10^

Literature shows a universal decline in TB screening and case-finding activities, as well as TB case notifications following the outbreak of the COVID-19 pandemic.^11,12^ This study shows that while efforts were made to continue TB services during the COVID-19 pandemic, significant gaps were noted. Evidence has shown that during the COVID-19 pandemic period, delays in TB diagnosis and treatment were noted because the focus was on containing the pandemic, yet the quality of TB care services was ultimately affected.^13,14^

During the COVID-19 pandemic, many TB clients experienced a high level of anxiety and depression, as they feared contracting COVID-19, interruptions to TB treatment and uncertainty surrounding the pandemic.^15,16^ Similarly, a study also demonstrated that the COVID-19 pandemic had a psychological effect on pulmonary TB patients in South Africa, while increased fear and anxiety were also observed in India because of the pandemic.^17^ Feelings of isolation following the lockdown and social distancing measures exacerbated the mental health issues of TB patients.

Zimbabwe, like other African countries, suffered the scourge of the pandemic. Several studies reported the stigma and discrimination that TB patients experienced during the COVID-19 pandemic, as they had to ask for authorisation letters to be allowed to travel to clinics for treatment. Adherence to TB treatment protocols remains an essential component of the TB programme in Zimbabwe^6,18^ and many other countries.

Conducted against a backdrop of increased evidence on the negative impact of the COVID-19 pandemic on TB service delivery,^12,19^ this study sought to explore the lived experiences of TB patients as they accessed and continued TB treatment during the COVID-19 pandemic period in Zimbabwe.

## Research methods and design

This study used an interpretative phenomenological analysis (IPA) design to explore the experiences of TB patients as they navigated treatment during the COVID-19 pandemic. Interpretative phenomenological analysis is a qualitative research method that focuses on understanding the lived experiences of the participants in the study.^20^ This research design is recognised for its capacity to explore participants’ perspectives in depth and extract meaningful insights.^20,21^ Additionally, Coyle and Murtagh suggest that researchers can derive meaning from participants’ thoughts, reflections and observations through this approach.^22^ Furthermore, IPA facilitates the interpretation of both the content and delivery of the participants’ responses.^23^ The design was selected for its suitability in exploring how individuals make sense of complex health experiences. Its focus on lived experience and making meaning aligns well with the study’s purpose of understanding TB patients’ treatment journeys during the COVID-19 pandemic in Zimbabwe. Methodological rigour was ensured through purposive sampling, following the IPA framework in analysis and reflexive journaling throughout the research process. In addition, the study was underpinned by Heideggerian (Interpretive) Phenomenology, which provides a philosophical foundation for exploring lived experiences through interpretation.

The research was conducted in Bulawayo, Zimbabwe’s second-largest city, in healthcare facilities that provide TB care services. The selected high-volume sites included Cowdry Park, Mzilikazi, Pelandaba, Maqhawe, Pumula South and Luveve, which offer comprehensive TB care services: screening, investigation and treatment. These clinics were chosen based on their high patient volume and significant TB burden. The participant recruitment was facilitated by nurses in charge of the selected clinics, who distributed information sheets, screening questionnaires and consent forms to potential participants on behalf of the researchers. The researchers collected the completed forms, conducted follow-up by telephone and scheduled interview sessions with the participants at the respective clinics telephonically. Purposive sampling was adopted to select TB patients who were accessing and continuing treatment during the COVID-19 pandemic period. A sample size of 15 participants was obtained, guided by the principle of data saturation, when no new information emerges.^24^ Data saturation was reached by the 13th participant, with two additional interviews conducted to confirm this saturation point.

The data collection took place from 15 April 2022 to 30 June 2022, following approval from the relevant authorities. The participants responded to a grand tour question: What were your experiences regarding accessing TB care services during the COVID-19 pandemic? In-depth telephonic interviews, which were audio-recorded, were conducted to collect the data. Examples of probing questions to elicit further insights are: How did you feel about accessing TB care services during the COVID-19 pandemic? What challenges did you encounter? and What recommendations would you make for future care? Each session lasted between 45 and 60 min. Because of concerns over the potential spread of COVID-19, telephonic interviews were selected as the preferred data collection method. Research has shown that telephone interviews are a practical and effective qualitative approach, facilitating access to geographically dispersed participants, ensuring a safe and comfortable interaction space and allowing scheduling flexibility.^25^

Although telephonic interviews limited the researchers’ ability to observe the participants’ non-verbal cues, they enhanced privacy and confidentiality.^26^ An interview guide was used to explore the participants’ experiences in accessing TB care services.

The data analysis adhered to the IPA framework, following the six-step process outlined by Smith and Osborn.^21^ Initially, one researcher transcribed the audio-recorded interviews verbatim. The analysis followed the six-step IPA framework. Initially, both researchers independently immersed themselves in the transcripts through repeated readings. They then annotated the texts and developed emerging themes. These themes were clustered into meaningful groups, leading to the creation of individual master tables comprising themes, subthemes and supporting excerpts. The researchers, together with an independent coder, compared their analyses to identify patterns and divergences. Finally, a master list of themes was developed through collaborative synthesis, ensuring analytical depth and coherence.

Consensus was reached between the researchers and the independent coder, resulting in a final table of themes. To ensure accuracy, the researcher who collected the data contacted 10 accessible study participants to review the thematic summary and confirm that their perspectives were appropriately represented. Once the participants validated the findings, the researchers finalised the thematic framework.

The researchers upheld trustworthiness in the study by ensuring credibility, confirmability, dependability and transferability, following the framework established by Lincoln and Guba.^27^

Credibility pertains to the accuracy of the findings. To enhance credibility, member checking (as alluded to above) was conducted to verify the accuracy and interpretation of the participants’ data. Confirmability was maintained through independent analysis by both the researchers and an experienced qualitative research coder. Dependability was reinforced through comprehensive documentation and detailed explanations of the research process, including the data collection and analysis. Transferability was facilitated by providing an in-depth description of the context of the study.

### Ethical considerations

The study adhered to the principles of the Declaration of Helsinki, which safeguards the rights of research participants.^28^ Ethical clearance to conduct this study was obtained from the Medical Research Council of Zimbabwe (No. MRCZ/A/2835). Permission to conduct the study was secured from the Director of City Health Services in Bulawayo. Prior to the interviews, all the participants voluntarily signed written informed consent forms, which were physically collected from the facilities.

Additionally, verbal consent was obtained before the telephone interviews. To protect the identities of the healthcare facilities involved in the study, alphabetical codes were assigned to each; the participants’ anonymity was ensured by using pseudonyms. The study adhered to the principles of respect, beneficence and justice. As the primary researcher, the first author acknowledges her dual role as both a nurse and a researcher and recognises that her prior experience with TB care services may have influenced the research process and interpretation of findings. To enhance reflexivity and minimise potential bias, she engaged in ongoing reflexive journaling and participated in peer debriefing throughout the study. These strategies supported critical self-examination and helped ensure that the participants’ voices remained central to the analysis.

## Findings

The participants were aged 21–63 years and had been accessing treatment from the sampled facilities for a period of 2–6 months. Table 1 presents the sociodemographic characteristics of the participants.

**TABLE 1:** Participants’ sociodemographic data.

No.	Pseudonym	Age (in years)	Gender	Employment status	Treatment facility	Period on TB treatment (months)
01	Lovemore	26	M	Unemployed	B	3
02	Endurance	38	M	Employed	A	2
03	Linda	23	F	Unemployed	B	4
04	Lee	53	F	Self-employed	C	3
05	Sibahle	45	F	Employed	A	6
06	Busi	62	F	Unemployed	C	5
07	Ntando	21	F	Unemployed	A	4
08	Sibonile	63	F	Unemployed	D	3
09	Bongani	39	F	Unemployed	B	2
D10	Buhle	46	F	Employed	B	6
D11	Musa	42	F	Self-employed	A	4
D12	Veli	39	F	Unemployed	C	3
D13	Thoko	40	F	Unemployed	A	5
D14	Thembi	35	F	Employed	C	3
D15	Suku	31	F	Unemployed	D	2

TB, tuberculosis.

The study yielded three themes: psychological effects, support systems and TB service delivery gaps. Each of the themes yielded subthemes, which are also presented in Table 2.

**TABLE 2:** Themes and subthemes.

Theme	Subtheme
1. Psychological effects	Increased anxiety and fear
	Unintended disclosure
	Stigma and discrimination
2. Support systems	Healthcare facility support
	Family and community support
3. TB service delivery gaps	Delays in TB diagnosis
	Follow-up and support
	Health education

TB, tuberculosis.

### Psychological effects

The TB clients reported that they had experienced psychological effects such as increased anxiety and fear, unintended disclosures, stigma and discrimination.

#### Increased anxiety and fear

The changes in the client flow at the clinic (having to wait outside or in the shed) affected the clients, particularly during the early days of the pandemic:

‘The thought of going to the clinic was a nightmare, having to wait outside even when it was cold, made me anxious.’ (Veli, female, 39 years old)

‘One morning when I got to the clinic with cough and chest pain, we were told that we should not get inside the clinic. We were given masks to put on and screening was done whilst we were standing outside. We were given information on COVID-19. Thereafter, I was told that I should go inside the clinic and sit in some corner waiting to be tested for COVID-19. I had a lot of anxiety and fear.’ (Thoko, female, 40 years old)

Some patients were hesitant to go to the healthcare facility because of the fear of contracting the disease. The following excerpts are illustrative of this:

‘I was hesitant and not interested in going to the clinic. I feared getting COVID-19.’ (Suku, female, 31 years old)

‘With some patients coughing at the clinic, it created more anxiety in me. I was afraid of contracting COVID-19 and taking it to my family.’ (Thembi, female, 35 years old)

#### Unintended disclosure

The clients had to be in possession of travel authorisation letters for them to be able to pass through police roadblocks to access TB medication. Alternatively, they had to produce their medical records as proof of going for a medical check-up.

Some participants had this to say:

‘I was only able to pass through a police roadblock after telling them that I was on TB treatment and showing them my medical records. This was the most frustrating time as I navigated my way to the clinic.’ (Sibonile, female, 53 years old)

‘Going to the clinic to collect my TB medicines was a real challenge, because the police required an authorisation letter to travel or some form of proof that I needed to go to the clinic. I had to carry my medicine containers with the few remaining tablets for me to be allowed to pass through the police roadblock. I did not like the thought of going to the clinic.’ (Ntando, female, 21 years old)

#### Stigma and discrimination

Some participants indicated that they experienced stigma and discrimination during the pandemic, as shown below:

‘Because I was coughing, family members were afraid that I could be having COVID-19. They made a conscious effort not to come close to me and would pass some funny comments.’ (Buhle, female, 46 years old)

‘I could hear comments from the neighbours and some family members talking about me that I possibly had COVID-19. I felt stigmatised and frustrated.’ (Bongani, female, 39 years old)

### Support systems

The participants in this study indicated that they received some form of support from the healthcare facility, family, community and friends. This support was perceived to have significantly contributed to their recovery.

#### Healthcare facility support

The extracts below show that participants felt supported at the healthcare facility:

‘The nurse I found at the clinic in the room where I collected my medication was encouraging and constantly reminding me that I should take my medication and keep my mask on. She shared her phone number and indicated I could call if I have challenges.’ (Musa, female, 42 years old)

‘The nurses were supportive, although we were told we should spend the shortest time at the clinic.’ (Lee, female, 53 years old)

#### Family and community support

Participants also received support from their families and communities, as shown by the following excerpts:

‘My family members were supportive, but I could see that they were afraid. My sister constantly called me checking if I was taking my TB treatment.’ (Sibahle, female, 45 years old)

‘My siblings were concerned about my health; my brother would send money for me to buy food.’ (Busi, female, 62 years old)

Friends and peers seemed to have played a key role in terms of a support system, as the following extracts illustrate:

‘My friends from church would pray with me through a WhatsApp call; I found that soothing and encouraging. I believe that contributed to my healing.’ (Lovemore, male, 26 years old)

‘I was able to make friends with someone [patient] I met at the clinic, shared our cell numbers and we constantly communicated through WhatsApp messages. That was a source of encouragement as we interacted with each other, we shared our concerns, jokes, and good news about getting better.’ (Endurance, male, 38 years old)

### Tuberculosis service delivery gaps during the coronavirus disease 2019 pandemic

Some of the clients’ narrated experiences indicated TB service delivery gaps such as delays in TB diagnosis, follow-up and support, as well as in health education.

#### Delays in tuberculosis diagnosis

About this gap, participants had this to say:

‘I was tested for COVID-19 and waited for the results that came after two weeks. Fortunately, the results were negative. But the cough persisted. I was then tested for TB. The results came a week later and were positive. It was only then that I started TB treatment.’ (Linda, female, 23 years old)

‘TB testing and a chest x-ray were done after I had visited the clinic several times.’ (Sibahle, female, 45 years old)

#### Follow-up and support

Community follow-up is a crucial component of TB service delivery. This component could not be done during the COVID-19 pandemic, as shown below:

‘I had been told at the clinic that the nurses and community workers would visit me at home and possibly check if other relatives had TB or not. However, this did not happen. Perhaps it was because of the COVID-19 pandemic.’ (Linda, female, 23 years old)

‘The community workers do a lot of support for people who are sick in the community; however, during the pandemic, they never visited me. I think they were afraid of contracting COVID-19.’ (Busi, female, 62 years old)

#### Health education

The health education emphasis was on COVID-19 rather than TB. Participants had this to say:

‘Health education was done on a daily basis on a number of health topics; however, the emphasis was on COVID-19.’ (Ntando, female, 21 years old)

‘Whilst I suffered from pulmonary TB, the nurses were concerned about COVID-19 and spoke more about it, how it was transmitted and how it could be prevented. They mentioned the issue of TB when they talked about how to take TB treatment.’ (Bongani, female, 39 years old)

## Discussion

The study explored the experiences of TB patients as they accessed and continued TB treatment during the COVID-19 pandemic in Bulawayo, Zimbabwe. The study reveals the changes in the processes of accessing healthcare services during the COVID-19 pandemic, which affected the participants’ emotional and psychological well-being. The pandemic introduced new infection control protocols and restrictions, such as requiring individuals to wait outside healthcare facilities in cold weather and wear face masks before being allowed inside. As part of these measures, participants were also instructed to sit in a corner away from other clients and nurses. These practices left many feeling fearful, stressed and anxious. These practices, though intended to minimise the risk of COVID-19 transmission, were perceived as dehumanising and contributed to feelings of rejection and vulnerability.

The findings of this study resonate with global evidence highlighting the psychological toll of the COVID-19 pandemic on both healthcare providers and patients. Huang’s study in China revealed a high incidence of anxiety and stress among medical staff and patients, primarily driven by the fear of contracting the disease.^29^ This fear not only increased emotional distress but also disrupted the delivery of healthcare services, discouraging patients from seeking care, including for conditions such as TB. Supportive evidence from other contexts further emphasises the widespread impact of the pandemic on TB patients. In addition, Mandal’s study in India reported psychological distress^17^ among individuals with pulmonary TB, while two South African studies by Scheunemann and Thungana confirmed that the mental health of TB patients was adversely affected by the pandemic.^15,30^ These findings collectively suggest that the convergence of COVID-19 and TB care created significant barriers to accessing services, with fear and anxiety acting as major barriers. This highlights the urgent need for healthcare systems to adopt more compassionate and psychologically informed approaches during public health emergencies.

Participants had unintended disclosures of TB status through police roadblocks requiring medical documentation or medicine containers as they navigated their way to healthcare facilities. This resulted in exposure to privacy violations and social stigma. Moyo noted similar findings in Zimbabwe for clients who needed to access HIV care services.^31^ This not only violated patient privacy but also exposed individuals to stigma and discrimination. The psychological toll of such disclosures was compounded by the social perception that coughing signalled COVID-19 infection, resulting in TB patients being shunned by family and community members. The intersection of TB and COVID-19 thus created a dual burden of stigma, intensifying feelings of isolation and fear.^32,33^

This study revealed varied psychological experiences among TB patients during the COVID-19 pandemic. While some participants faced stigma and discrimination, others found strength and support from family, community members and fellow patients. These sources of emotional and financial support were crucial for coping and treatment adherence, echoing findings by Daftary, Loveday and others who emphasised the value of peer and familial support in TB care.^34,35^ However, contrasting evidence from Vanleeuw and Kim highlighted increased vulnerability because of income loss and disrupted support systems during lockdowns, which intensified psychological distress.^36^ The dual burden of TB and COVID-19 compounded fears of stigmatisation and social anxiety, as noted by Kim and other scholars.^37,38^ Service delivery was also affected. Participants reported delays in TB diagnosis and reduced follow-up care, consistent with studies by Di Gennaro, Gandhi, Téllez-Navarrete and Aznar.^11,13,14,39^ These disruptions were attributed to the healthcare system’s prioritisation of COVID-19 at the expense of other diseases, for example, TB. Although telehealth was recommended by Shen and the WHO to maintain continuity of care, its implementation was limited in this context.^3,40^

Additionally, participants faced significant logistical barriers, such as transport disruptions and police roadblocks, which not only limited their physical access to healthcare services but also had profound psychological effects. These challenges contributed to feelings of helplessness, frustration and heightened vulnerability, compounding the emotional burden already associated with TB and the COVID-19 pandemic.

This qualitative study offers a rich, experiential understanding of how the pandemic affected TB service delivery, particularly from the perspective of TB care nurses. Unlike previous quantitative studies in Zimbabwe that primarily documented service disruptions, this study brings to light the lived realities behind those statistics for studies in Zimbabwe.^41,42^ It reveals how systemic changes such as lockdown measures, resource reallocation and altered care protocols resulted in personal struggles, emotional distress and, in some cases, remarkable resilience and adaptation.

These findings contribute to a growing body of evidence, including studies by Thekkur et al., which highlight the multifaceted impact of the pandemic on TB control efforts.^7^ By centring the voices of those directly affected (patients and frontline healthcare workers), this study emphasises the critical importance of empathetic, context-sensitive approaches to healthcare delivery, especially during times of crisis.

### Implications

The study highlights the urgent need for resilient, patient-centred TB care services capable of withstanding a crisis. Policy measures should ensure uninterrupted access to TB services through emergency preparedness while integrating psychosocial support to address stigma, anxiety and unintended disclosure. Protecting patient confidentiality and promoting health education are essential to correct misinformation. On a practical level, healthcare facilities must improve client flow, strengthen community-based support and maintain TB awareness during health crises.

### Recommendations

To ensure continuity of TB services during public health emergencies such as the COVID-19 pandemic, it is essential to adopt resilient and integrated strategies such as telehealth and other community-based approaches.

Evidence from best practices demonstrates that aligning TB and COVID-19 services, coupled with robust community partnerships and close monitoring, can mitigate disruptions in care.^3,40^ It is such innovative strategies that ministries of health can adopt for similar emergencies. Gains made from containment of public health diseases, such as TB, should be preserved and not reversed by the emergency of pandemics like COVID-19.

Integrating TB services into emergency preparedness frameworks further ensures sustained access to care in future crises. It is in the interest of the ministries of health to adopt the use of digital health tools, such as SMS reminders and telehealth consultations, to ensure treatment adherence and positive treatment outcomes, particularly when in-person visits are restricted.

Implementing psychosocial support systems, if adopted during emergencies, can assist in addressing stigma and mental health challenges associated with overlapping TB and COVID-19 burdens.

## Conclusion

The study revealed that TB patients in Zimbabwe faced significant psychological distress during the COVID-19 pandemic, including anxiety, fear, stigma and unintended disclosure of their health status. Despite these challenges, many found strength in support systems from healthcare providers, family, friends and communities. However, gaps in TB service delivery included delays in diagnosis, reduced follow-up and limited TB-specific health education. Such scenarios demonstrate a strain in the already fragile healthcare system. These findings emphasise the need for resilient, patient-centred TB care that integrates psychosocial support and maintains service continuity during public health crises.
